# Evolution and spread of a highly drug resistant strain of *Mycobacterium tuberculosis* in Papua New Guinea

**DOI:** 10.1186/s12879-022-07414-2

**Published:** 2022-05-06

**Authors:** Arnold Bainomugisa, Evelyn Lavu, Sushil Pandey, Suman Majumdar, Jennifer Banamu, Chris Coulter, Ben Marais, Lachlan Coin, Stephen M. Graham, Philipp du Cros

**Affiliations:** 1 Queensland Mycobacteria Reference Laboratory, Brisbane, QLD Australia; 2 University of Papua New Guinea, Port Moresby, Papua New Guinea; 3 Central Public Health Laboratory, Port Moresby, Papua New Guinea; 4 Burnet Institute, 85 Commercial Road, Melbourne, VIC 3004 Australia; 5 University of Melbourne Department of Paediatrics and Murdoch Childrens Research Institute, Royal Children’s Hospital, Melbourne, VIC Australia; 6 University of Sydney, Sydney, NSW Australia; 7 Peter Doherty Institute, Melbourne, VIC Australia

**Keywords:** Drug resistance, Mycobacterium tuberculosis, RR-TB, Whole genome sequencing, Papua New Guinea

## Abstract

**Background:**

Molecular mechanisms determining the transmission and prevalence of drug resistant tuberculosis (DR-TB) in Papua New Guinea (PNG) are poorly understood. We used genomic and drug susceptibility data to explore the evolutionary history, temporal acquisition of resistance and transmission dynamics of DR-TB across PNG.

**Methods:**

We performed whole genome sequencing on isolates from Central Public Health Laboratory, PNG, collected 2017–2019. Data analysis was done on a composite dataset that also included 100 genomes previously sequenced from Daru, PNG (2012–2015).

**Results:**

Sampled isolates represented 14 of the 22 PNG provinces, the majority (66/94; 70%) came from the National Capital District (NCD). In the composite dataset, 91% of strains were Beijing 2.2.1.1, identified in 13 provinces. Phylogenetic tree of Beijing strains revealed two clades, Daru dominant clade (A) and NCD dominant clade (B). Multi-drug resistance (MDR) was repeatedly and independently acquired, with the first MDR cases in both clades noted to have emerged in the early 1990s, while fluoroquinolone resistance emerged in 2009 (95% highest posterior density 2000–2016). We identified the presence of a frameshift mutation within Rv0678 (p.Asp47fs) which has been suggested to confer resistance to bedaquiline, despite no known exposure to the drug. Overall genomic clustering was significantly associated with *rpoC* compensatory and *inhA* promoter mutations (p < 0.001), with high percentage of most genomic clusters (12/14) identified in NCD, reflecting its role as a potential national amplifier.

**Conclusions:**

The acquisition and evolution of drug resistance among the major clades of Beijing strain threaten the success of DR-TB treatment in PNG. With continued transmission of this strain in PNG, genotypic drug resistance surveillance using whole genome sequencing is essential for improved public health response to outbreaks. With occurrence of resistance to newer drugs such as bedaquiline, knowledge of full drug resistance profiles will be important for optimal treatment selection.

**Supplementary Information:**

The online version contains supplementary material available at 10.1186/s12879-022-07414-2.

## Background

The global emergence and spread of drug-resistant tuberculosis (DR-TB) is threatening global efforts to eliminate the disease [1]. In 2020, *Mycobacterium tuberculosis* (MTB) strains that are multi-drug resistant and rifampicin resistant TB (MDR/RR-TB) accounted for an estimated 465,000 incident cases globally, but only 150,359 were registered on treatment [1, 2]. Extensively drug resistant TB (XDR-TB), which is MDR/RR-TB with additional resistance to a fluoroquinolone and at least one other group A drug [3] poses a dire public health threat.

Papua New Guinea (PNG) is one of the high burden countries for both TB and MDR-TB, with an estimated incidence of 441 and 22 per 100,000 population respectively [1]. A population survey carried out in four provinces of PNG estimated the MDR-TB prevalence in 2014 to be 2.7% and 19.1% among new and previously treated cases respectively, and 593 laboratory confirmed MDR/RR-TB cases were notified in 2020 [1, 4]. There are several recognized MDR/RR-TB hotspots in PNG including National Capital District (NCD) and South Fly District, Western Province [4, 5]. Of MDR-TB isolates cultured from 2010 to 2017, 2.6% were also resistant to a fluoroquinolone [5]. Understanding the epidemiology and emergence of DR-TB is important to inform public health efforts in controlling the disease within PNG, but also for neighbouring countries such as Indonesia and Australia, with cross-border transmissions recently reported [6]. Further, the potential fitness cost of drug-resistant mutations to decrease the transmissibility of drug-resistant compared to drug-susceptible strains may have an important epidemiological impact.

There are limited studies of bacterial factors as key drivers of DR-TB prevalence and persistence in PNG. Prior analysis of clinical isolates from Daru, a town in Western Province that is located approximately 500 km from Port Moresby in the NCD, provided critical insight of MTB population dynamics and molecular causes of drug resistance [7]. With a modern Beijing highly resistant strain identified to be responsible for the majority of DR TB in Daru, the potential spread of this strain to other PNG settings needed to be investigated. Furthermore, knowledge provided by whole genome sequencing (WGS) of the prevalent mutations associated with drug resistance is important to inform recommendations for the choice of effective treatment regimens. There have been major changes in recent years to the recommendations by the World Health Organization (WHO) for the treatment of MDR/RR-TB, with a welcome shift to shorter and safer, injectable-free regimens when possible [8]. An improved understanding of the drug-resistance patterns of MTB strains in PNG will inform the development of national guidelines for PNG and improve treatment outcomes.

In this study, we aimed to use WGS on MDR/RR-TB clinical isolates from PNG to understand the genomic features pertaining to acquisition of drug resistance and reconstruction of drug resistance evolution within the dominant strain in this dataset. We also expanded the analyses to include previous WGS dataset from Daru, PNG to investigate evolutionary history, clonal relatedness, acquisition of resistance and compensatory resistance at a wider scope.

## Methods

### Study population and specimen selection

We retrospectively sampled RR-TB clinical isolates referred from the Central Public Health Laboratory (CPHL), Port Moresby, PNG to the Supra-National Reference Laboratory, Brisbane, Australia. The criterion for referral is detection of rifampicin resistance by Xpert MTB/RIF assay (Cepheid, Sunnyvale, USA) on concurrent samples or at the individual discretion of a clinician. Using a sample collection from a 24-month period (03 January 2017 to 21 January 2019), every second isolate was selected for study inclusion. Solid cultures on Lowenstein Jensen media rather than clinical specimens (as in the Daru study) were the preferred specimens referred to the Supra-National Reference Laboratory for culture and drug susceptibility testing (DST). To include a broader phylogenetic perspective, raw WGS and DST data of 100 MTB isolates from the previously published Daru study [7] were included to form a composite dataset for analysis.

### Drug susceptibility testing

DST was performed for five first-line drugs (isoniazid, rifampicin, ethambutol, streptomycin, pyrazinamide), and seven second-line drugs (ofloxacin, kanamycin, capreomycin, amikacin, ethionamide, para-aminosalicylic acid and cycloserine), as described previously [6, 7].

### Whole genome sequencing

MTB isolates were retrieved from − 80 °C storage, cultured on Lowenstein–Jensen medium, and DNA extracted using an organic enzymatic method [7]. WGS was performed at Forensic and Scientific Services, Brisbane, Australia, with Illumina Technology (HiSeq 2500) using Nextera XT library preparation kits, as instructed by the manufacturer (Illumina, San Diego, CA). Paired end fastq files (raw sequencing data) were submitted to NCBI Sequence Read Archive (Project no. PRJNA757443, Additional file 1: Table S1 for accession numbers). Obtained reads were quality checked using FastQC (version 0.11.2, http://www.bioinformatics.babraham.ac.uk/projects/fastqc), trimmed using trimmomatic version 0.27 [9], and mapped to *M. tuberculosis* H37Rv (NC_000962.3) using BWA-MEM [10]. Alignments were refined using Samtools [11] and GATK [12] toolkits, with regard to base quality re-calibration and alignment corrections for possible polymerase chain reaction artifacts. GATK UnifiedGenotyper was used to call single-nucleotide polymorphisms (SNPs) and small insertion/deletions (indels), and they were annotated using SnpEff [13]. Variants with a minimum read depth of 10 (5 reads in both forward and reverse orientation), with Phred score > 30, < 0.6 strand bias and > 75% allele frequency were utilized for downstream analysis. Variants within repetitive gene regions such as PPE/PE and consecutive variants within a 10 bp window flanking indels were excluded.

### Genotypic drug resistance prediction

The annotated variant file for each isolate was assessed for presence of mutations in all genes known to confer resistance to the different anti-TB drugs, including regulatory genes (Additional file 2: Table S2). The identified mutations were further evaluated using Integrative Genomic Viewer (IGV) to view the quality of reads mapping at the genomic position of the mutation for each isolate. The presence of each mutation was also screened for in the ReSeqTB drug resistance database [14]. Mutations that were not clearly linked to phenotypic drug resistance were reported as genotypic non-wild type and were not considered as genotypic resistance markers but, in some instances where an unknown mutation causes a frameshift in a target gene, they were considered as plausible resistance markers. When no mutation was detected in the relevant target genes, the isolate was considered to be phenotypically susceptible. Mutations in genes coding for the RNA-Polymerase subunits *rpoA*, *rpoB* (excluding resistance mediating mutations in the rifampicin resistance determining region (RRDR), and in codons 170, 400, 491), and *rpoC* were reported as putative fitness compensating variants for RR strains as suggested previously [15, 16].

### Phylogenetic and molecular clock inference

MTB lineage and sub-lineage SNP typing was performed according to Coll et al. [17]. For a conservative and robust phylogenetic reconstruction, mutations in genes known to confer resistance and bacterial fitness were excluded, except known phylogenetic markers in drug resistance genes as per Merker et al. [18] (Additional file 2: Table S3). Using concatenated SNP alignments, initial phylogenetic analysis was performed by ModelFinder software [19], implemented by IQ-Tree [20] to infer an optimal substitution model for the alignments. Maximum likelihood phylogenetic trees were constructed using IQ-Tree, applying the generalized time-reversible model with four gamma categories for rate variation, ascertainment bias on, and 1000 ultrafast bootstrap replicates [21]. Phylogenies were rooted with the midpoint rooting option using FigTree v1.4.2. To investigate the phylogenetic placement the dominant Beijing strain in PNG in relations to the global context, a global sequence data collection of Beijing lineage (Study accession number PRJEB7281) was used for this purpose [22].

To reconstruct a timed phylogeny for the dominant strain in this dataset using Beast 1.8.2 [23], we first assessed the temporal signal in the alignment by testing the correlation of date of sampling and root-to-tip distance, using TempEst v1.5. Beast template files were created using BEAUTI, applying informative uniform prior distribution on substitution rate as previously reported [24, 25], a coalescent constant size demographic model, the Hasegawa–Kishino–Yano substitution model with four gamma categories, and Markov-Chain-Monte-Carlo (MCMC) chain length of 70 million (10% burn-in) with sampling of every 10,000 traces/trees. Using an XML-input file that has been manually modified to specify the number of invariant sites, Bayesian Evaluation of Temporal Signal was used to further evaluate the best molecular clock. In this approach, analyses with strict and relaxed molecular clocks with and without the tip dates were performed to find the best supported clock for our data. We then ran different demographic models (i.e. coalescent constant size, exponential, and Bayesian skyline) under a relaxed molecular clock using tip dates and the same parameters for the site model and MCMC as described above. We calculated Bayes factors from marginal likelihood estimates obtained from path sampling and stepping stone sampling among the models to get the best. Inspection of BEAST log files with Tracer v1.6 was used to check adequate mixing of the Markov chains and assess whether all parameters observed had Effective Sample Size > 200, suggesting an adequate number of effectively independent draws from the posterior sample and thus sufficient statistical support. TreeAnnotator v2 was used to obtain the best supported topology under the maximum clade credibility method.

To assess the changes of the effective population size within the Beijing strain in PNG, Bayesian skyline plot was calculated using Beauti in-put parameters of the best coalescent model. We further used a random starting tree, a chain length of 70 million (10% burn-in) and collected 5000 traces/trees. Again, adequate mixing of the Markov chains and Effective Sample Size values in the hundreds were observed. A maximum clade credibility genealogy was calculated with TreeAnnotator v2. Variants considered as drug resistance markers and putative compensatory mutations were analyzed individually, and mapped on the phylogenetic tree to define variant diversity and drug resistance evolution within the cohorts.

### Transmission cluster

Using SNP distance matrix generated using Ape package in R and sampling dates for the specimens, we utilized two clustering approaches integrated in TransCluster software to compare their results since our dataset was composed of two data cohorts from different time frames, which can affect SNP accumulation and transmission processes within the algorithm. Transmission approach is based on sample pairs clustering together if the estimated transmission events between them are lower than a threshold number, at a given probability. The transmission clusters were to portray not only recent direct transmission events within the study population, but also earlier transmission events that are connected by contacts that are not sampled.

### Statistical analysis

Statistical analyses were done in R [26]. We compared terminal branch lengths from the molecular clock tree (measured as the number of SNPs mapped to each terminal branch) between Daru and non-Daru Beijing isolates using the 2-sample Kolmogorov–Smirnov test [27]. Fishers exact test was used to assess both genomic and patient characteristics in the dataset.

## Results

### Study population, selection and patient characteristics

**Fig. 1:**
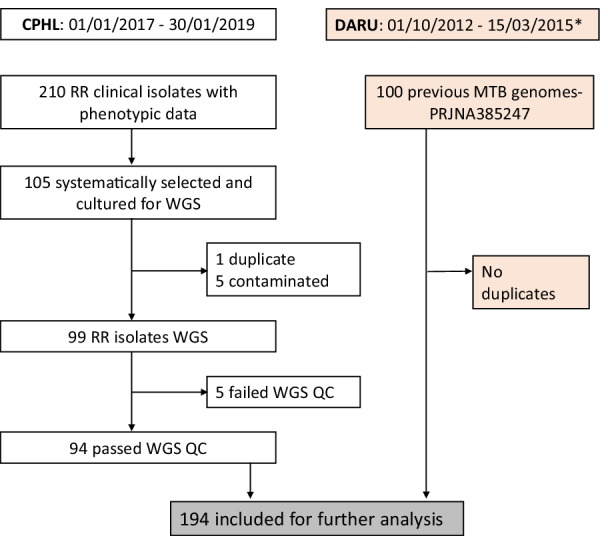
Sample selection for whole genome sequencing. *CPHL* Central Public Health Laboratory, *MDR-TB* Multi-drug resistant tuberculosis, *WGS* Whole genome sequencing, *QC* Quality control. *Daru strains represented all cultured isolates over the study period; no selection

**Fig. 2:**
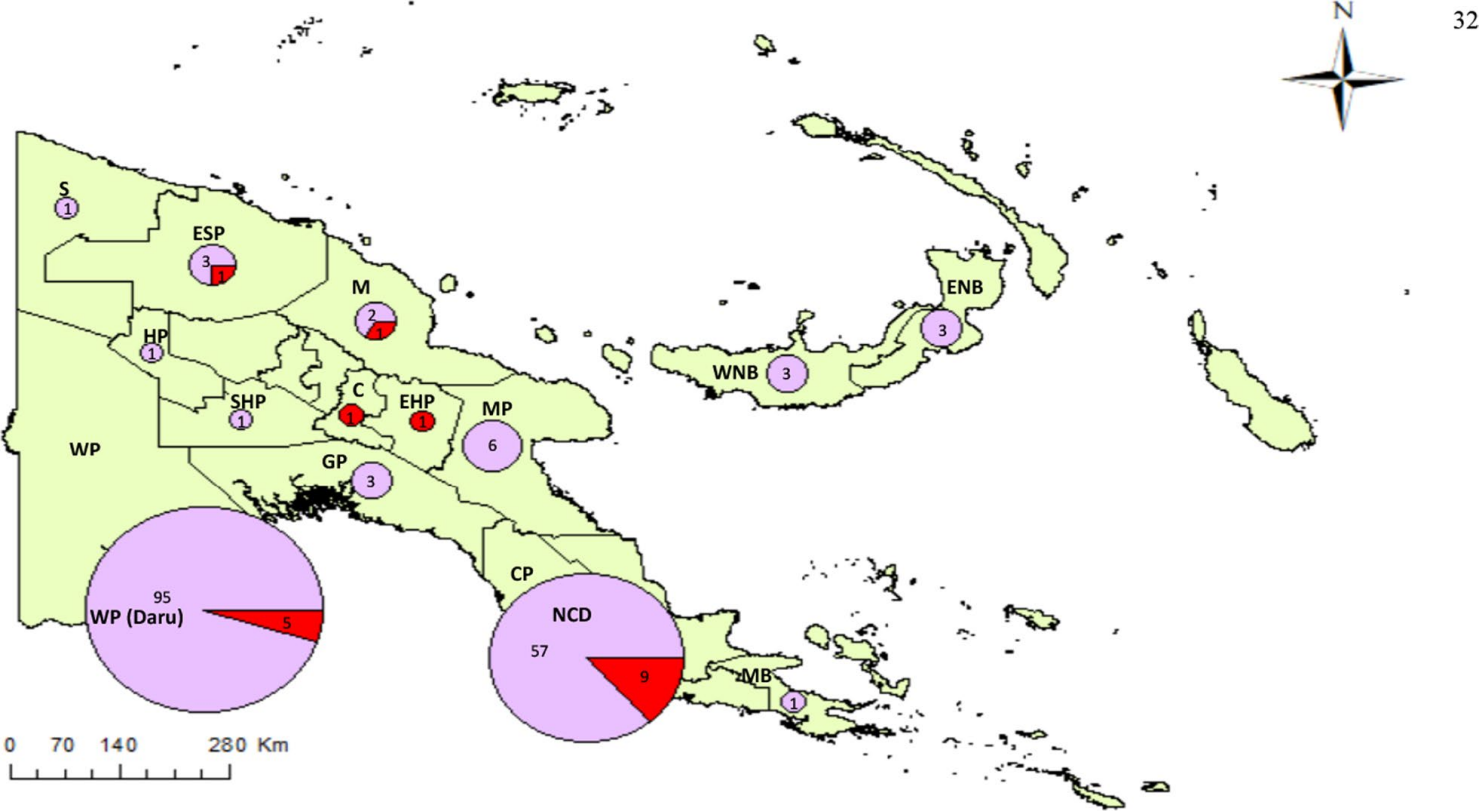
Distribution of *Mycobacterium tuberculosis* lineages identified in the study population, with strains collected from various provinces of Papua New Guinea (L2—Purple, L4—Red). Provinces include *S* Sandaun, *ESP* East Sepik Province, *M* Madang, *HP* Hela Province, *WP* Western Province, *SHP* Southern Highland Province, *EHP* East Highland Province, *GP* Gulf Province, *C* Chimbu, *MP* Morobe Province, *CP* Central Province, *NCD* National Capital District, *MB* Milne Bay, *WNB* West New Britain, *ENB* East New Britain

A total of 210 rifampicin resistant clinical isolates were referred from CPHL during January 2017 to January 2019, and culture-based susceptibility results data were identified. Each patient had one isolate and data about their previous TB exposure were not available. After systematic sampling, 105 of these isolates were selected for WGS and 94 (89%) were successfully available for analysis (Fig. 1). The additional selection of 100 clinical isolates from 165 MTB isolates referred in 2012–2015 from Daru, a town in Western Province that is located approximately 500 km from Port Moresby in the NCD, led to a composite dataset of 194 isolates. Besides Western Province, the CPHL isolates were from clinical samples from patients in 14 of the 22 geographical provinces of PNG, with the majority from NCD province (66/94, 70%) (Fig. 2). Most patients were young adults with sputum smear-positive pulmonary TB, with no significant differences between known characteristics of patients from NCD and Daru (Table 1).

**Table 1:** Characteristics of patients from National Capital District and Daru cohorts

Characteristic	NCD (n = 66)	Daru (n = 100)	p value
Lineage			
L2 (Beijing 2.2.1.1)	57 (86%)	95 (95%)	0.084
L4 (Euro-American mainly 4.8)	9 (14%)	5 (5%)	
Age			
< 18	6 (9%)	16 (16%)	0.246
≥ 18	58 (88%)	82 (82%)	
Unknown	2 (3%)	2 (2%)	
Median (IQR)	33 (25–38)	29 (20–35)	
Gender			
Male	30 (48%)	48 (48%)	0.754
Female	36 (52%)	52 (52%)	
Sputum smear			
Positive	17 (26%)	80 (80%)	0.981
Negative	4 (3%)	20 (20%)	
Unknown (Inc. Isolates)	45 (68%)	0	
Type of TB			
Pulmonary*	63 (95%)	99 (99%)	0.302
Extrapulmonary	3 (5%)	1 (1%)	
Genomic cluster**			
Yes	53 (66%)	87 (87%)	0.278
No	13 (34%)	13 (13%)	
Phenotypic resistance to first-line drugs			
HR	17 (26%)	0	-
HRE	5 (8%)	0	
HRS	14 (21%)	24 (24%)	
HRZ	3 (5%)	0	
HRES	7 (11%)	5 (5%)	
HRSZ	3 (5%)	6 (6%)	
HREZ	3 (5%)	0	
HRESZ	14 (21%)	50 (50%)	
Nil	0	15 (15%)	

*NCD* National Capital District, *IQR* inter-quartile range, *TB* tuberculosis; *H* Isoniazid, *R* Rifampicin, *E* Ethambutol, *S* Streptomycin, *Z* Pyrazinamide

^*^Predominantly sputum samples; extrapulmonary samples rarely collected

^**^Genomic cluster—determined from transmission cluster analysis with pairwise SNP difference of 10 between isolates

### MTB population structure and susceptibility testing

Utilizing WGS analysis on the dataset, 3321 SNPs and 574 small indels were identified. SNP typing using Coll et al. classification [17] and phylogeny using IQTree (Additional file 2: Fig. S1) revealed 176 isolates to be of lineage 2 (sensu stricto modern Beijing sub-lineage 2.2.1.1) and the rest were of lineage 4 (Euro-American 4.8, mainly T, n = 18). As reported previously from Daru, the Beijing strain was dominant in almost all observed provinces of PNG (Fig. 2), including as the only strain from East New Britain (n = 3), Gulf (n = 3), Hela (n = 1), Milne Bay (n = 1), Morobe (n = 6), Sandaun (n = 1), Southern Highlands (n = 1) and West New Britain (n = 3). Majority of strains observed at NCD were also of the Beijing lineage (86%, 57/66), while all three isolates from neighbouring Chimbu and Eastern Highlands provinces were lineage 4 strains.

Analysis of pairwise SNP difference revealed a median of 29.21 (IQR 10–41) and 318 (IQR 104–489) among lineages 2 and 4 respectively. Comparison of SNP difference among Beijing strains from NCD and Daru revealed a significant difference (median 20.3-Daru vs 28.1-NCD, mood’s median test p < 0.001), highlighting the close relatedness within Daru strains.

We compiled a dataset comprising of all Beijing genomes from PNG and 110 Beijing genomes from 28 different countries [22] representing all the five global regions. Using 6920 high quality SNP to create an alignment, phylogenetic analysis revealed PNG strain formed a monophyletic clade (Additional file 2: Fig. S2), within region of difference 150 (RD150) group and the nearest neighbouring clade constituted genomes from mainly Kiribati, Indonesia, Vietnam, Thailand, Germany, Burma, Eswatini and Zimbabwe.

Phenotypic susceptibility testing of all 94 isolates and the previous Daru dataset (100 isolates) showed that 15 (8%) were fully susceptible to all first-line drugs, and these were all from Daru. All 85 MDR/RR isolates from Daru had resistance to an additional first-line drug. All isolates from CPHL were MDR/RR, and MDR without resistance to additional first-line drugs was found in 17 (28%) of 66 isolates from NCD and 11 (39%) of 28 from other provinces (Table 1). Combined resistance to all five first-line drugs (HRESZ) was significantly higher in Daru (50%) compared to CPHL (14%), p < 0.001. There were 15 pre-XDR (MDR/RR with additional fluoroquinolone resistance) isolates, 12 from Daru, two from NCD and one from Hela province. In Table 2, we present the percentage of genotypic susceptible strains by clade according to updated WHO TB drug grouping [28].

**Table 2:** Genotypic susceptibility of *Mycobacterium tuberculosis* strains in NCD, Daru, and other PNG provinces

WHO Group	Anti-TB drug	% NCD strains susceptible (n = 66)	% Daru strains susceptible (n = 100)	% Other province strains susceptible (n = 28)
Group A	Levofloxacin OR moxifloxacin	95	88	96
	Bedaquiline	98	100	100
	Linezolid	100	100	100
Group B	Clofazimine	98	100	100
	Cycloserine OR terizidone	100	100	100
Group C	Ethambutol	52*	44*	71*
	Delamanid	100	100	100
	Pyrazinamide	61*	43*	75*
	Imipenem-cilastatin OR meropenem, with clavulanic acid	Unknown		
	Amikacin	98	98	100
	Streptomycin	36	12	42
	Ethionamide OR prothionamide	38*	14*	46*
	Para-aminosalicylic acid	98	100	98
–	High dose Isoniazid	27*	33*	29*

*NCD* National Capital District, *PNG* Papua New Guinea, *TB* Tuberculosis

^*^Concern about decreased susceptibility to TB drugs included in the WHO approved all-oral short course RR/MDR-TB drug regimen

### Evolutionary history of Beijing strain and acquisition of drug resistance

Assessment of the temporal signal within the data using TempSt revealed a linear correlation (Additional file 2: Fig. S3, R^2^ = 0.384) between sampling time and root-to-tip distance which supports use of relaxed molecular clock. Further evaluation of the molecular clock using Bayesian Evaluation of Temporal Signal, where analyses were run with strict and relaxed molecular clocks with and without the tip dates, found Bayes factor supported relaxed molecular clock for our data (Additional file 2: Table S4). To assess phylogeny, divergence times and evolutionary rates of the dominant strain in this dataset based on 1101 SNPs, Beast runs identified exponential, Hasegawa–Kishino–Yano substitution model as the best supported model (Additional file 2: Table S5). Mutation rate was estimated to be 0.45 SNPs/genome/year (95% highest posterior density [HPD] 0.34–0.52), which is similar to other published studies [22, 25, 29].

**Fig. 3:**
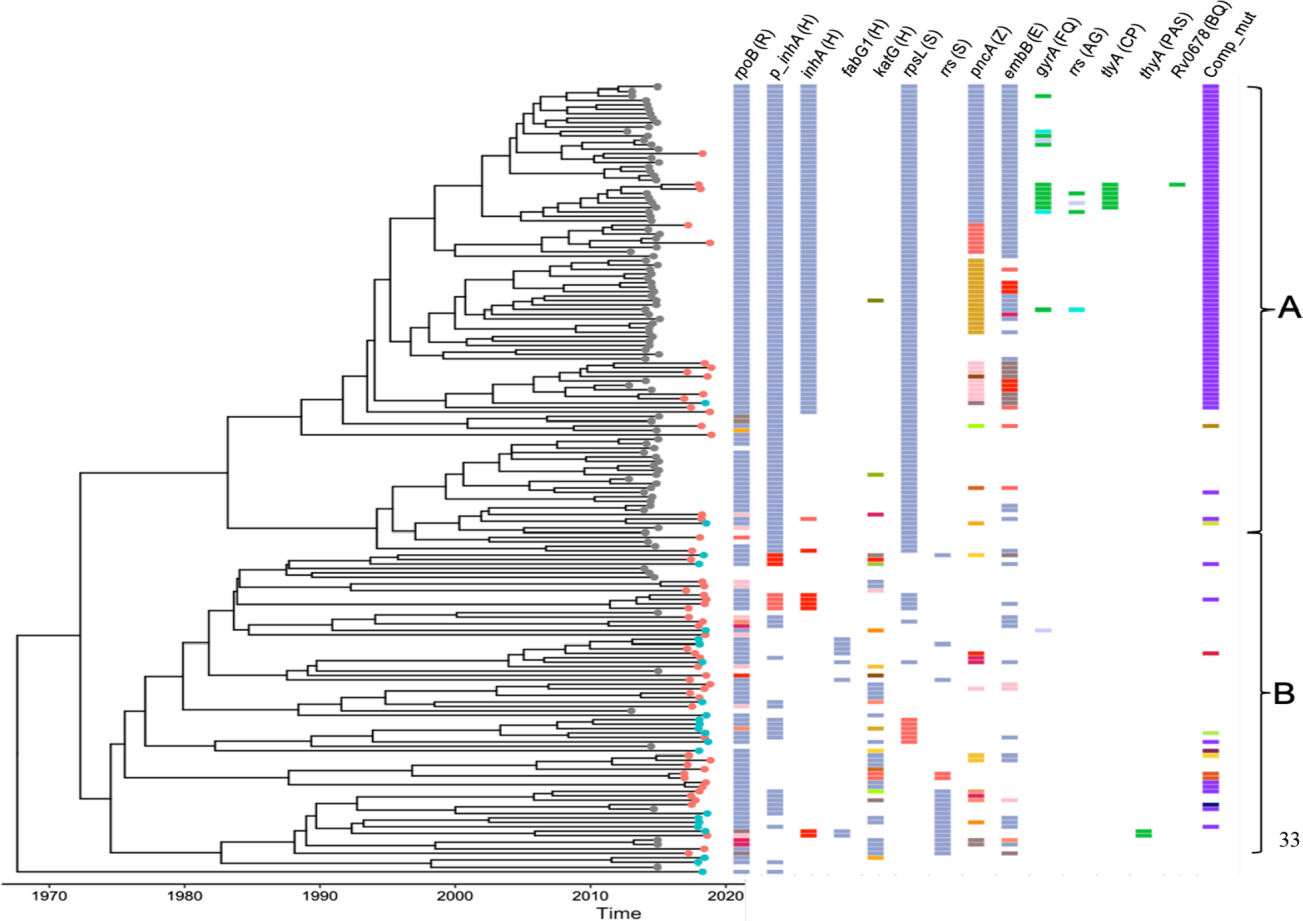
Phylogenetic tree with inferred timeline demonstrating the evolutionary history of 176 MTB (sensu stricto Beijing L2.2.1.1) strains identified in different Papua New Guinea provinces. Phylogeny constructed using the best evolutionary model (Hasegawa–Kishino–Yano substitution, relaxed clock, exponential demographic) revealing two clades (A-Daru dominant clade, B-NCD dominant clade), with tip labels colors; grey—Daru, orange—National Capital District, green—other provinces. Columns show drug resistance associated mutations for first- and second-line drugs (different mutations per column represented by different colors), and putative compensatory mutations in the RNA polymerase genes *rpoA* and *rpoC*.; *R* Rifampicin, *H* Isoniazid, *S* Streptomycin, *Z* Pyrazinamide, *E* Ethambutol, *FQ* Fluoroquinolone, *AG* Aminoglycosides, *CP* Capreomycin, *PAS* Para-aminosalicyclic acid, *BQ* Bedaquiline, *Comp_mut* Rifampicin compensatory mutations

According to the tree topology, there was a tendency for the strains to form two major clades, Daru dominant clade-A and NCD dominant clade-B (Fig. 3). Of the 104 strains in Daru dominant clade (83 from Daru), 19 were from NCD, 1—Hela province and 1—East New Britain province, while only 12 Daru strains were identified among the NCD dominant clade (n = 72), indicative of limited strain migration and dispersion between Daru and other settings. However, majority of strains from other provinces (n = 22) were identified among clade B, which highlights considerable strain overlap and dispersion between NCD and other provinces of PNG.

**Fig. 4:**
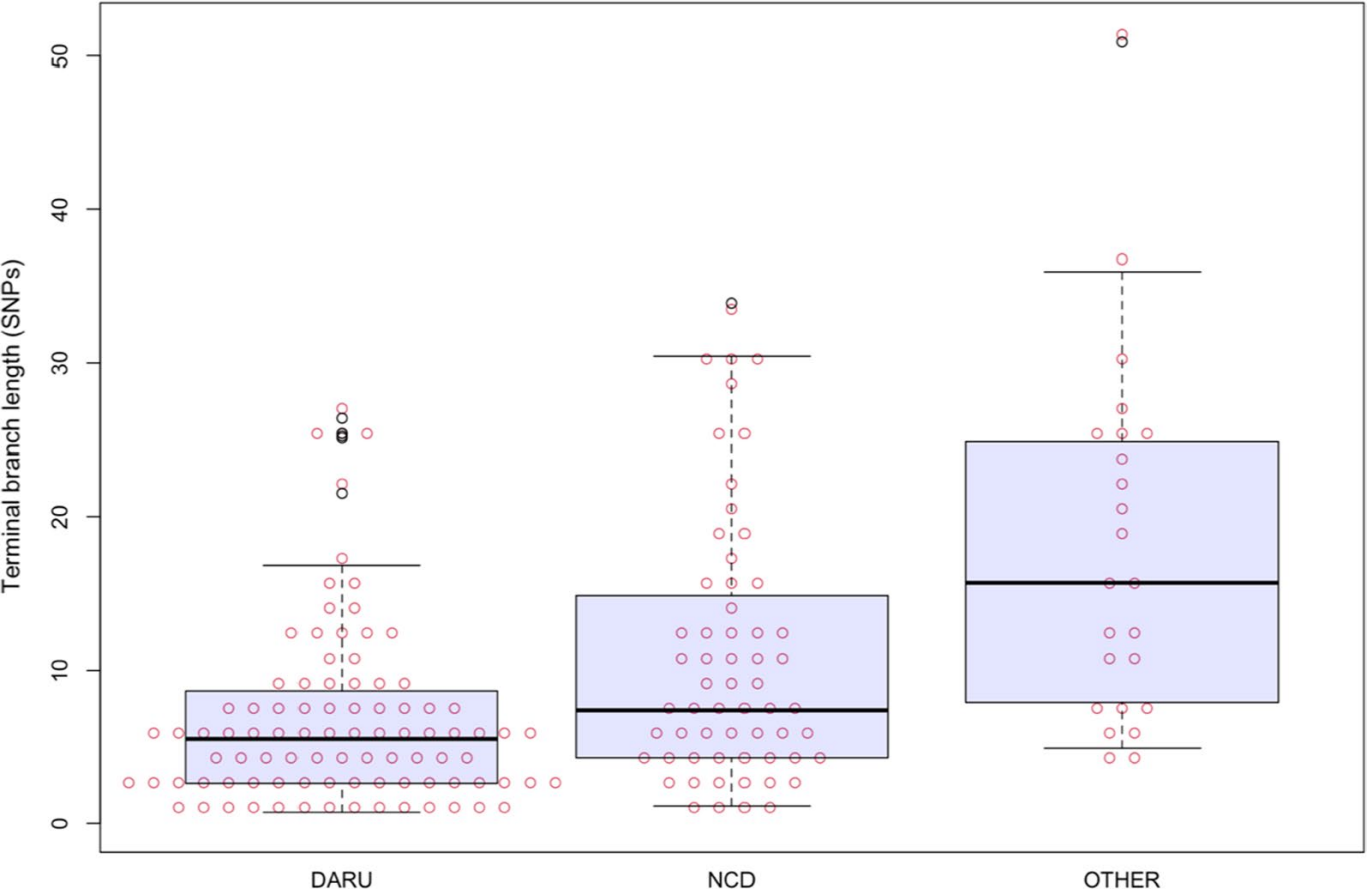
Terminal branch lengths for a Beast-estimated phylogenetic tree of Beijing strains identified at Daru, NCD (National Capital District) and other provinces of PNG

With the time to the most recent ancestor (TMRCA) for the entire dominant strain in this dataset was determined to have emerged around 1967 (95% HPD 1959–1976), the Daru dominant clade had TMRCA of 1983 (95% HPD 1977–1988) compared to 1972 (95% HPD 1966–1979) for NCD dominant clade. This highlights that the Beijing strain was transmitted in other settings earlier than its introduction in Daru and not vice versa. Using 2-sample Kolmogorov–Smirnov test and considering differences in sample sizes, terminal branch lengths were significantly shorted among Daru isolates (Fig. 4; mean terminal branch length: 5.72) when compared to NCD isolates (mean terminal branch length: 8.21, p = 0.007) and to isolates from other provinces (mean terminal branch length: 15.48, p < 0.001). In this context, short terminal branch length is indicative of higher relative rates of transmission than with long terminal branches.

In order to gain more detailed insights into the emergence of resistance mutations in the evolutionary history of the Beijing strain, we parsimoniously mapped mutations on the temporal phylogeny (Additional file 2: Fig. S4). As in the previous Daru paper [6], we determined a shared TMRCA for Streptomycin (*rpsL* p.Lys43Arg) and Isoniazid (*fabG1-inhA* C-15T) resistance to be 1983 (95% HPD 1977–1988) among the Daru dominant clade, while *rrs* A514C (mediate Streptomycin resistance) first emerged in the progenitor among NCD dominant clade in 1988 (95% HPD 1983–1993), and then first acquired MDR status in 1992 (95% HPD 1987–1996) through both *fabG1-inhA* T-8C (mediate isoniazid resistance) and *rpoB* p.Ser450Leu mutations (mediate rifampicin resistance). While TMRCA for MDR-TB status among Daru dominant clade was estimated to be 1993 (95% HPD 1988–1997) through step-wise acquisition of *rpoB* p.Ser450Leu, the same mutation was earlier observed in 1987 (95% HPD 1980–1995) among NCD dominant clade before attaining MDR status. Interestingly, we observed five different clones where rifampicin resistance conferring mutations precede acquisition of isoniazid resistance conferring mutations, highlighting the need for further scale up of rapid diagnostics and improved treatment management.

**Fig. 5:**
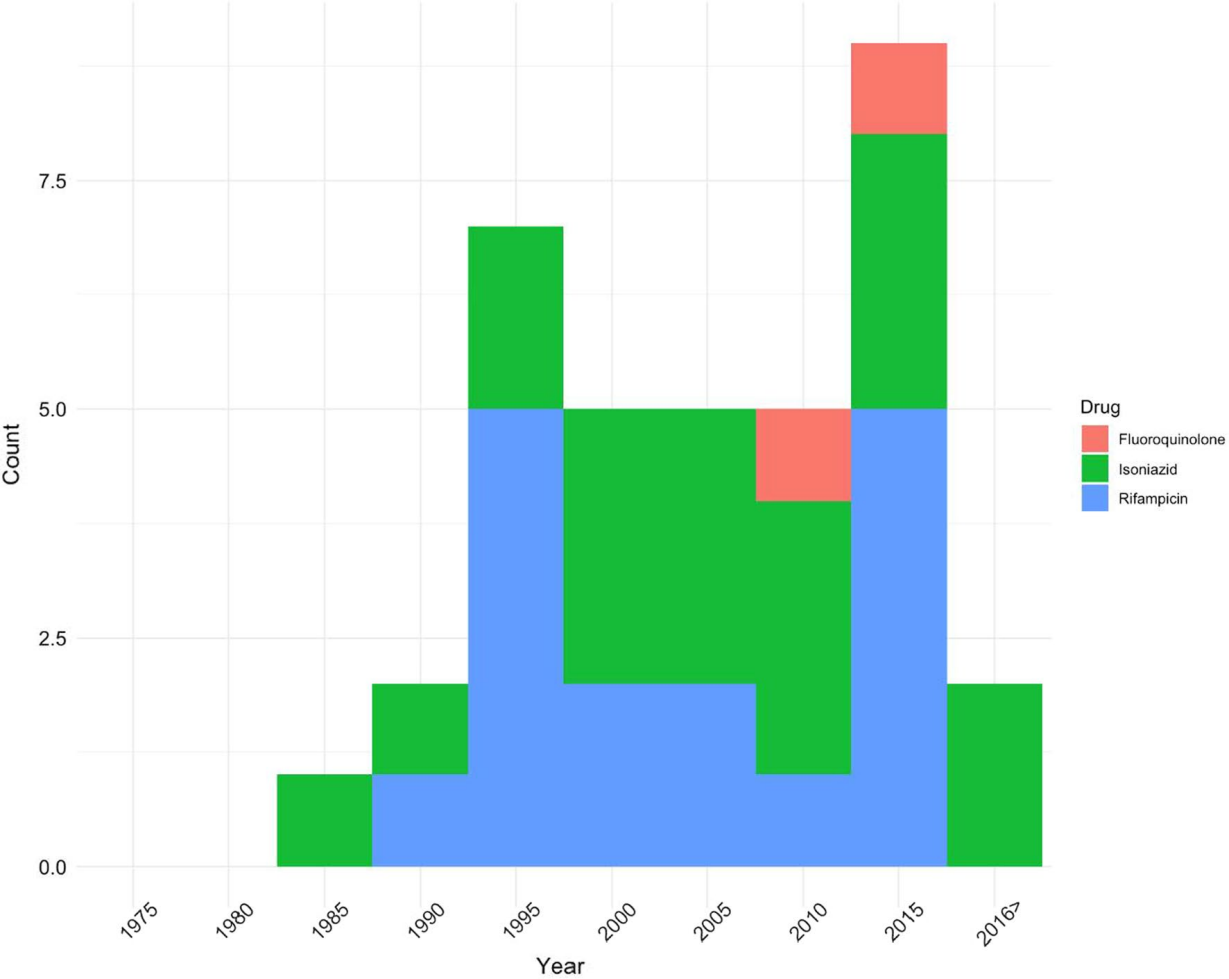
Independent emergence of resistance among Beijing 2.2.1.1 over time based on the age of nodes where resistance mutations were inferred to have emerged (Additional file 2: Fig. S4). Mutations on the terminal branches weren’t considered

Fluoroquinolone resistance, mediated through *gyrA* mutations, were all recently acquired. TMRCA for clones identified in 2009 (95% HPD 2000–2016) and 2013 (95% HPD 2008–2018) was through p.Asp94Gly mutation shared among 6 and 2 isolates respectively in Daru dominant clade, illustrating primary transmission of fluoroquinolone resistance. No clones with fluoroquinolone resistance were observed among the NCD dominant clade. Nodes on the phylogenetic tree (ignoring mutations on terminal tree branches) indicate MDR-TB clone emergence between early 1980s to mid 1990s (Fig. 5), with fluoroquinolone resistant clones emerging more recently in 2009.

Generally, we observed MDR and fluoroquinolone resistance emerging repeatedly, frequently and independently within the dominant Beijing strain in this dataset. The order in which the Beijing strain acquired drug-resistance mutations is consistent with both treatment guidelines in place at the time (that patients receive a fluoroquinolone only after they are found to have rifampicin-resistant TB) and the historical timeline of when each corresponding antibiotic was globally introduced for clinical use.

### Drug resistance mutations and their diversity among Beijing strains, CPHL

There was good overall correlation between genotypic and phenotypic susceptibility testing, with only ethambutol and pyrazinamide showing discrepancy (Additional file 2: Fig. S5). Only 58% (19/33) of strains with putative ethambutol resistance conferring mutations were phenotypically resistant at critical concentration (5.0 μg/ml); minimum inhibitory concentration (MIC) testing at lower concentrations was not performed. The majority of resistance conferring mutations were in *embB* gene; however, there were two different ethambutol resistant isolates with *embA* upstream mutations, one with C-16A co-existing with p.Gln497Pro while the other had only C-16T. Resistance to isoniazid demonstrated the greatest diversity of resistance conferring mutations within four known genes—*katG*, *fabG1-inhA*, *inhA,* and *fabG1*.

Isoniazid is a pro-drug that requires activation by a multifactional and essential catalase peroxidase (*katG*) gene. We identified 10 and 8 known *katG* mutations among strains from NCD and other provinces respectively, responsible for resistance in 34/81 (42%) of isoniazid resistant isolates (Additional file 2: Fig. S6). Nineteen (55%) of these mutations were due to a change on the conventional codon 315 (p.Ser315Thr—n = 17 and p.Ser315Asn—n = 2), and the rest were singlet mutations on various *katG* codons that included frameshift (p.Asn529fs n = 1) and *katG* promoter (A-10C). We also noted three isoniazid resistant isolates with multiple point mutations within *katG*, p.Phe185Leu-p.Val68Ala, p.Ser315Thr-p.Arg563Leu and p.Asn138Ser-p.Gly548Ala-p.Arg643Leu and all resistant at 0.4 μg/ml isoniazid critical concentration.

Of the 81 isoniazid resistant isolates at low level critical concentration, 58% (47/81) had mutations within the *fabG1-inhA* promoter region, of which 85% (40/47) were C-15T, 9% G-17T (4/47) and 6% T-8C (3/47). Of the isolates with *fabG1-inhA* promoter mutations, only 47% (17/47) were susceptible at isoniazid high level critical concentration hence may be effectively treated at higher dose. We also explored the co-occurring mutations among isoniazid resistant isolates, of the 22 isolates with mutations in *inhA* open reading frame (p.Ile21Val, n = 14; p.Ile21Thr, n = 1; p.Ile194Thr, n = 7), 20 had occurrence with *fabG1-inhA* promoter mutations (C-15T, n = 16; G-17T, n = 4) and 14 *ndh* frame-shift mutation (del.G304). Surprisingly, we found 8 isoniazid resistant isolates with *fabG1* silent/synonymous mutation, p.Leu203Leu which confers resistance through formation of alternative promoter thereby increasing the transcriptional levels of *inhA* [30]. These mutations were distributed across the CPHL clade on the tree, which reveals the ongoing selective pressure (convergent evolution) on isoniazid resistance. With all isoniazid resistant isolates tested for ethionamide resistance, all isolates with *fabG1-inhA* promoter mutations were ethionamide resistant though one isolate had co-occurrence with unknown *ethA* mutation, p.Thr44Ala.

Resistance to rifampicin was identified in all isolates, with seven different mutations within the RRDR. Although most mutations were observed on codon 450 (n = 69), there were three different alleles of mutations on codon 450; p.Ser450Leu (n = 67), p.Ser450Ala (n = 1) and p.Ser450Thr (n = 1). We also observed a rare phenomenon of 2 less frequently observed mutations (p.Gln432Leu and p.Asp435Gly) within the RRDR of one isolate. Overall, 37% (30/81) of all Beijing strains at CPHL carried putative compensatory mutations in *rpoC* (n = 29) and *rpoA* (n = 1), which is less than what was observed among isolates from Daru (66%, p = 0.001). The mutation *rpoC* p.Val483Gly accounted for 66% (20/30) of Beijing isolates with putative compensatory effects.

There was also high diversity in *pncA* mutations for pyrazinamide resistance among isolates from NCD (n = 10 different mutations), compared to 6 in Daru and 5 in other provinces. We noticeably identified three phenotypically pyrazinamide susceptible isolates with rare *pncA* mutations, p.Gln10Pro (n = 2) and p.Ile31Thr. Interestingly, two pyrazinamide resistant isolates had rare *pncA* promoter mutation, *pncA-Rv2044c,* A-11C, which highlights the ongoing selective pressure for further spread of pyrazinamide resistance in this population.

### XDR genotype and putative second-line resistance markers

One isolate was genotypically identified to be XDR (MDR-TB resistant to fluoroquinolone and at least additional group A drug) by resistance to bedaquiline with a frameshift mutation (p.Asp47fs) within *Rv0678*, which putatively confers resistance to bedaquiline [31]. Phenotypic DST was not performed and there was no prior exposure to bedaquiline in the patient. Besides the *ndh* frame shift mutation (p.Glu102fs) putatively thought to confer resistance to clofazimine, there were no known mutations observed in genes that confer resistance to other newer/re-purposed TB drugs.

**Fig. 6:**
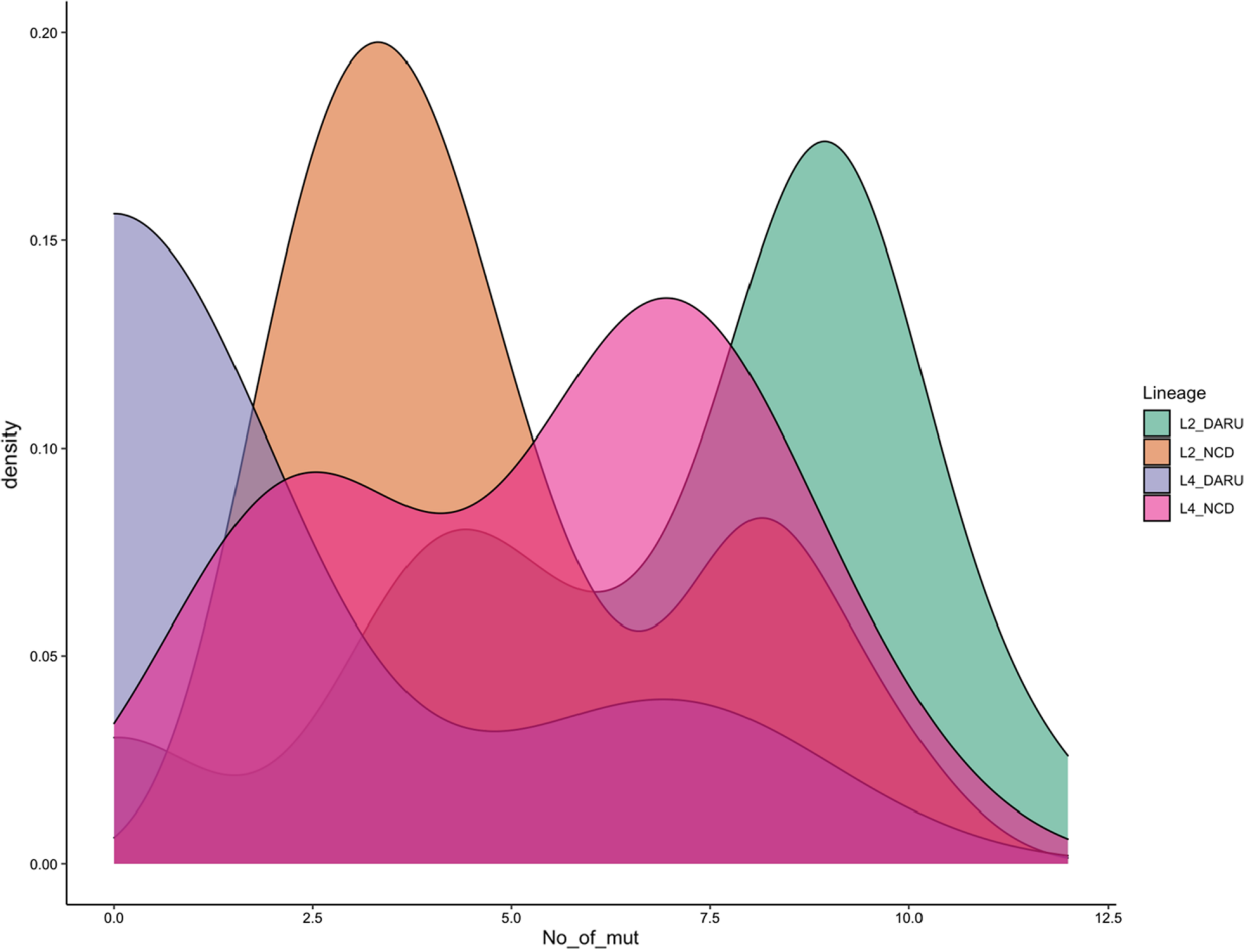
Density plot of the number of resistance-conferring mutations (exclude compensatory mutations), stratified by lineages for all isolates from Daru and National Capital District (NCD) provinces

We also identified two isolates with nonsense mutation (p.Gln111X) within *thyA* gene, known to confer resistance to para-aminosalicylic acid (PAS). Interestingly, no known prior exposure of these patients to PAS was established, though the isolates shared a common phylogenetic node and had four SNP difference between themselves despite coming from different provinces (NCD and Morobe). Overall in the composite dataset, enumeration of known resistance conferring mutations per patient (excluding compensatory mutations) revealed patients with Lineage 2 strain from Daru had a significantly higher average number of mutations than patients from NCD (7.1-Daru vs 4.5-CPHL, p = 0.001, Fig. 6).

### Transmission clusters

Using a transmission threshold of 21 and transmission rate of 2, 14 pairs or case genomic clusters were identified among all L2 isolates (82%, 145/176) and 1 cluster among the L4 (62%, 11/18); which was equivalent to using a maximum of 10 differentiating SNPs between at least two isolates (SNP threshold). Fourteen genomic clusters were formed among 145 Beijing strains, 60% (87/145) were from Daru, 31% (46/145) were from NCD, while the rest were from other provinces (Additional file 2: Fig. S7). Only 7/14 genomic clusters constituted strains from different provinces, with the largest cluster (B) having 104 strains from four different provinces (83-Daru, 18-NCD, 2-East New Britain and 1-Hela) while cluster M was the most diverse, which highlights plausible intra-transmissions of the strain across provinces.

Interestingly, 12/14 clusters involved at least one strain from NCD, which highlights the fact that strains from NCD are very widely dispersed. Using genomic clusters as a variable to infer transmission of the dominant strain within this dataset, we assessed the contribution of two common genomic features such as *rpoB* compensatory mutations and *inhA* promoter mutation (*fabG1-inhA*) towards transmission. Both features were found to significantly increase the transmission of TB, (p < 0.001, Additional file 2: Table S6).

## Discussion

The use of WGS and analysis of a composite dataset from two different study periods and geographical locations, reveal the evolutionary history of drug resistance acquisition among the dominant strain in this dataset, Beijing L2.2.1.1 in PNG. Relative to a global Beijing collection, PNG Beijing isolates were found to be essentially monophyletic, closely related to strains from South Asian, European and African countries which illustrates the global spread of this strain. Geographically, this strain was found in multiple PNG settings in addition to NCD and Daru, providing evidence of disease spread across PNG, likely to be driven by human migration. Previously recognized to be responsible for the clonal expansion of MDR-TB in Daru [6], its identification in other provinces suggests that MDR in PNG is driven by a few clades from this strain. Determination of when and where the MDR outbreak began assists in identification of priority areas for public health surveillance and understanding networks underlying geographical dispersion of MDR. We estimate that the Beijing strain attained the first MDR phenotype in the early 1990s in areas of NCD province. This was more than 3 years before the major Daru MDR outbreak, indicating that the strain emerged years before it was identified by existing clinical and public health surveillance activities. This is supported by early TMRCA (mid 1970s) for the NCD dominant clade and longer branch length when compared to Daru dominant clade. It is noteworthy that the dominant strain in this dataset remained chronic in NCD and other provinces for a long time without causing a major outbreak as occurred in Daru. The ‘comb-like’ structure of the tree, apparent in the distal portion of the Daru-dominant clade, suggests a highly infectious clone.

Temporal reconstruction of the resistance mutation acquisition among Beijing strains showed contemporaneous early acquisition of streptomycin (*rpsL*) and low-level isoniazid resistance among Daru dominant clade in the early 1980s, which illustrates that low-level isoniazid resistance is a sentinel event heralding development of MDR-TB among Daru isolates. Klopper et al. recently reported *inhA* promoter mutations to not be a gateway to XDR-TB, but to also have a compensatory role in isoniazid resistance considering that they rarely occur in absence of other resistance mutations [32]. We observed similar findings though occurrence of *inhA* promoter mutation in the absence of other isoniazid conferring mutations was not rare. Further investigation into the role of *inhA* promoter mutations in a background of ethionamide and high-level isoniazid resistance is needed. In contrast, in NCD dominant clade, occurrences of rifampicin resistance preceding isoniazid resistance were observed, caused by variety of mutations within *katG*, *inhA* and *inhA* promoter mutations. This difference in MDR-TB acquisition between NCD and Daru may be explained by effects of differing TB treatment options. However, there is limited literature about the availability of treatment regimens in different settings on PNG at the time. Figure 5 not only summarizes the inferred timing of individual resistance emergence events from the early 1980s to mid 2010s, it also shows how MDR-TB emerged repeatedly and independently across provinces, with emergence of pre-XDR in the later years. This calls for swift action using the best-informed interventions available to efficiently tackle the growing challenge of increased development and transmission of resistant strains.

Of particular interest is the high diversity of resistance conferring mutations to first line drugs among NCD isolates when compared to Daru isolates. Isoniazid resistance conferred by *katG* mutations had highest (55%) variability, similar to previous descriptions in other countries such as Mexico (53.7%) [33], Nepal (57.8%) [34], Brazil (56%) [35] and Morocco (59%) [36]. High mutation variability correlates with occurrence of convergent evolution [37], which illustrates the strong evidence of positive selection for isoniazid resistance. This is supported by evidence of homoplasy through a rare *fabG1* silent/synonymous mutation (p.Leu203Leu), which causes isoniazid resistance by increasing the transcriptional levels of *inhA* [30]*.* A previous study indicated *katG* mutation, p.Ser315Thr to have negligible fitness cost [38], and probably contributes to the low spread of MDR strains as compared to Daru dominant clade which has *inhA* promoter mutation (C-15T). Although there was significant difference between the number of *rpoB* compensatory mutations present at Daru and NCD, presence of both *inhA* promoter and *rpoB* compensatory mutations were found to be significantly associated with MDR clusters. These findings highlight how pathogen-specific factors alone may not fully explain the differential success of DR-TB transmission in Daru and NCD. Interestingly, 12 of 14 genomic clusters involved NCD isolates, which highlights existence of multiple clones of the strain in the same locality. This implicates NCD to be an amplifier of MDR transmissions to other provinces and vice versa, since it’s the central hub for influx of people from other provinces. There is a need to further investigate the prevailing TB transmission dynamics within NCD, including understanding a range of factors such as socio-economics, host genotypes and bacterial genotype, for better implementation of the national TB response plan.

Interestingly, we identified a clonal acquisition of *ndh* frame-shift mutation (p.Glu102fs) in only the Daru dominant clade, in presence of *inhA* promoter mutation that arose before the *ndh* mutation. Mutations in the *ndh* gene, encoding an NADH dehydrogenase, have previously been shown to cause defeats in enzyme activity, that resulting in increased NADH/NAD^+^ ratio and co-resistance to isoniazid and ethionamide [39, 40]. Since the molecular action of a re-purposed TB drug, clofazimine relies on the enzymatic reduction by the *ndh* gene to yield reactive oxygen species [41], we hypothesize the observed frame-shift mutation might elevate the MICs to clofazimine. While there’s a possibility that this mutation might have arisen by chance, unofficial literature shows that clofazimine was used in 1970/1980s for leprosy treatment in different settings in PNG. We hypothesize that co-infection (TB and leprosy) or misuse for leprosy treatment might explain the early acquisition and deeply rooted evolution of the resistance marker. Further, we identified a frame-shift mutation (p.Asp47fs) in Rv0678 gene, and mutations in this gene have previously been shown to cause cross-resistance between clofazimine and bedaquiline [42, 43]. A recent study in South Africa that included a sub-population of DR-TB patients found that a similar mutation, at baseline or emergent during treatment, was associated with poor clinical outcomes [31]. The bedaquiline resistant strain identified in our study raises concern surrounding the empiric treatment of DR-TB, even when constructed with novel or re-purposed agents. Individualized treatment regimens should be guided by WGS to achieve optimal treatment outcomes in all patients and prevent emergence of resistance to novel or re-purposed agents especially in high burden settings. Variations between provinces of susceptibility patterns to such a critical second-line drug as a fluoroquinolone challenges the utility of a national standard approach. The introduction of a diagnostic such as Xpert XDR and/or WGS could therefore facilitate adaptability of guidelines.

In Table 2, we present the 2018 WHO treatment guidelines [28] and show the percentage of patients that would benefit from each drug from Daru and NCD dominant clades. Though very low numbers of isolates were detected with fluoroquinolone resistance within both settings, a low proportion of patients would be eligible to receive one of the currently used shorter MDR-TB regimens in PNG (4–6 amikacin–moxifloxacin–clofazimine–prothionamide–pyrazinamide-high dose isoniazid-ethambutol/5 moxifloxacin–clofazimine–pyrazinamide–ethambutol) or WHO recommended shorter bedaquiline-containing all-oral regimen (4–6 bedaquiline (6 m)–levofloxacin/moxifloxacin–clofazimine–prothionamide–pyrazinamide–high-dose isoniazid–ethambutol/5 levofloxacin/moxifloxacin–clofazimine–pyrazinamide–ethambutol). This is largely due to the high proportion of resistance to thioamides (62%—NCD, 86%—Daru), high level resistance to isoniazid (73%—NCD, 67%—Daru) and common resistance to ethambutol. As per definition of the WHO exclusion criteria, for example any confirmed or suspected resistance to one drug (except isoniazid) in the short regimen, only 60%% (39/66 at NCD) and 43% (43/100 in Daru) of the patients infected with either a Beijing or L4 strain would be eligible for a shortened MDR-TB therapy. Based on common mutation profile, patients from both NCD and Daru are likely to benefit from a regimen composed of group A drugs and group B drugs. However, in a few cases, cross resistance to bedaquiline and clofazimine may necessitate the addition of group C drugs such as delamanid. Only two isolates were identified with known genomic marker (*thyA*, p.Gln111STOP) for PAS resistance [44], showing this may still be a useful drug for salvage regimens in PNG.

The study had a number of limitations. Not all notified cases had positive cultures for transportation to the reference laboratory which might have impacted on the number of isolates available for study selection. Provinces outside known MDR/RR-TB hotspots may potentially be under-testing with Xpert MTB/RIF potentially resulting in a bias of the isolates detected and included within the study that may not be fully representative of the MDR/RR-TB epidemic in PNG. However, all provinces had access to GeneXpert machines during the study period, and proportions of RR-TB from total tested were much lower in the provinces than in NCD [5]. In addition, not all isolates during the defined period were included due to funding limitations, however using every second isolate systematically reduced the risk of selection bias. Furthermore, use of isolates collected during different timeframes might have introduced study biases, considering that all the settings were experiencing different TB burden during those times. However, the long timeframe of the composite dataset allowed us to accurately estimate the evolution of drug resistance among the dominant strain within the dataset. Another limitation was the lack of detailed epidemiological data including disease importation or internal immigration, which limited our ability to precisely assess TB transmission across provinces. However, we were able to comment on the general patterns of TB transmission across provinces through use of genomics. Finally, as culture was only performed for samples that test positive on Xpert MTB/RIF, some RR-TB mutations may be missed, and mono-resistance to other drugs such as isoniazid were not detected and able to be included in the study.

## Conclusions

In conclusion, WGS analysis of the largest TB collection from PNG has enabled us to identify some of the plausible biological mechanisms contributing to the MDR transmission in PNG. There was evidence of resistant MTB strains across provinces and differential resistance markers for the dominant strain among isolates from NCD and Daru were inferred. Importantly, the short-course MDR-TB regimen recently endorsed by WHO is unlikely to be the most effective regimen choice for patients in NCD and Daru. In order to successfully control MDR-TB epidemics, access to rapid and comprehensive drug susceptibility testing, best supported by more advanced technologies like WGS and field diagnostics such as Xpert XDR, is crucial for improving surveillance and guiding individualized treatment to maximize cure and prevent further resistance acquisition.

## Supplementary Information

**Additional file 1:Table S1**. Drug susceptibility testing data and accession numbers.

**Additional file 2: Table S2.** *M. tuberculosis* genes associated with resistance or compensatory mutation. **Table S3.** Phylogenetic markers in known drug resistance genes as per Merker et al 2020. **Figure S1**. Phylogeny of 194 isolates collected from Daru (blue tip labels) and CPHL, Papua New Guinea. **Figure S2**. Phylogeny of 176 lineage 2 PNG isolates (purple - monophyletic clade) with a global collection of 110 Beijing genomes. **Figure S3**. Linear regression analysis showing correlation between root-to-tip distance and sampling years (R^2^ = 0.384) of an extended collection of 176 PNG Beijing datasets covering the period 2012 to 2019. **Table S4.** Bayesian evaluation of temporal signal (BETS) results for strict and relaxed molecular clock analysis of Beijing isolates. **Table S5.** Path and stepping stone sampling results of relaxed clock model selection based on marginal likelihood considering 100 steps and 10 million chain length. **Figure S4.** Evolutionary history of drug resistance among the Beijing strains of PNG forming two clades. **Figure S5.** Concordance of phenotypic and genotypic drug resistance among Beijing strains from CPHL (Green—bars) and Daru (Red—bars). **Figure S6.** Number of unique mutations within some of the common genes known to confer resistance to First-line drugs (*embB*—Ethambutol; *fabG1*, *fabG1-inhA*, *inhA*, *katG*—Isoniazid; *pncA*—Pyrazinamide; *rpoB*—Rifampicin) and Fluoroquinolone (*gyrA*). **Figure S7.** Genomic clusters created by Transcluster software (transmission threshold—21 and transmission rate—2) among Beijing strains, stratified by identified PNG provinces. **Table S6.** Association of genomic clustering among Beijing strains and two genomic features thought to be risk factors of transmission.

## Data Availability

Fastq files (raw sequencing data) for all strains analysed in this study are available from the Sequence Read Archive No. PRJNA757443 (www.ncbi.nlm.nih.gov/bioproject/PRJNA757443/), and details can be found within Additional file 1: Table S1.

## Abbreviations

CPHL: Central Public Health Laboratory
DR-TB: Drug resistant tuberculosis
DST: Drug susceptibility testing
HIV: Human immunodeficiency virus
HRESZ: H = isoniazid R = rifampicin E = ethambutol S = streptomycin Z = pyrazinamide
HPD: Highest posterior density
MCMC: Markov-Chain-Monte-Carlo
MDR: Multi drug resistance
MIC: Minimum inhibitory concentration
MTB: *Mycobacterium tuberculosis*
NADH/NAD+: Nicotinamide adenine dinucleotide hydrogen and its oxidised form
NCD: National Capital District
PNG: Papua New Guinea
Pre-XDR: Pre-extensively drug resistant TB
PAS: Para-aminosalicylic acid
RRDR: Rifampicin resistance determining region
RR-TB: Rifampicin resistant Tuberculosis
SNP: Single-nucleotide polymorphism
TB: Tuberculosis
TMRCA: Time to the most recent ancestor
WGS: Whole genome sequencing
XDR: Extensively drug resistant
WHO: World Health Organisation
