# Supplementary material for: Evolution and spread of a highly drug resistant strain of Mycobacterium tuberculosis in Papua New Guinea
Source: BMC Infect Dis. 2022 May 6;22:437. doi: 10.1186/s12879-022-07414-2 (PMC9077924; doi:10.1186/s12879-022-07414-2)
Supplement: Supplementary file 2 — Additional file 2: Table S2. M. tuberculosis genes associated with resistance or compensatory mutation. Table S3. Phylogenetic markers in known drug resistance genes as per Merker et al 2020. Figure S1. Phylogeny of 194 isolates collected from Daru (blue tip labels) and CPHL, Papua New Guinea. Figure S2. Phylogeny of 176 lineage 2 PNG isolates (purple - monophyletic clade) with a global collection of 110 Beijing genomes. Figure S3. Linear regression analysis showing correlation between root-to-tip distance and sampling years (R2 = 0.384) of an extended collection of 176 PNG Beijing datasets covering the period 2012 to 2019. Table S4. Bayesian evaluation of temporal signal (BETS) results for strict and relaxed molecular clock analysis of Beijing isolates. Table S5. Path and stepping stone sampling results of relaxed clock model selection based on marginal likelihood considering 100 steps and 10 million chain length. Figure S4. Evolutionary history of drug resistance among the Beijing strains of PNG forming two clades. Figure S5. Concordance of phenotypic and genotypic drug resistance among Beijing strains from CPHL (Green—bars) and Daru (Red—bars). Figure S6. Number of unique mutations within some of the common genes known to confer resistance to First-line drugs (embB—Ethambutol; fabG1, fabG1-inhA, inhA, katG—Isoniazid; pncA—Pyrazinamide; rpoB—Rifampicin) and Fluoroquinolone (gyrA). Figure S7. Genomic clusters created by Transcluster software (transmission threshold—21 and transmission rate—2) among Beijing strains, stratified by identified PNG provinces. Table S6. Association of genomic clustering among Beijing strains and two genomic features thought to be risk factors of transmission. [file 12879_2022_7414_MOESM2_ESM.docx]

**Table S2: *M. tuberculosis* genes associated with resistance or compensatory mutation.**

| **Drug** | **Genes** | **Publication no.** |
| --- | --- | --- |
| Rifampicin | *rpoB, rpoC, rpoA, rpoD, Rv2752c* | 1,3,6,7 |
| Isoniazid | *fabG1-inhA, inhA, katG,ndh, furA, oxyR, aphC, fadE24, srmR, kasA, mshA, Rv1258c, Rv2752c* | 1,3,7,8 |
| Ethambutol | *embB, embC, embA, ubiA, embR, iniA, iniC, manB, ubiA* | 1,6,7,8 |
| Pyrazinamide | *pncA, rpsA, panD, clpC1, rpsA, clpC1,* *Rv1258c, PPE35* | 1,6,7,8 |
| Streptomycin | *rpsL, gidB, rrs, whiB7, Rv1258c* | 1,6,7,8 |
| Ethionamide | *fabG1-inhA, ethA, ethR* | 1,6,7,8 |
| Fluoroquinolones | *gyrA, gyrB* | 1,6,7,8 |
| Amikacin | *rrs, whib7, gidB* | 1,4,8,11 |
| Kanamycin | *Rv2417c-eis, whiB7, rrs, gidB* | 1,4,8,10,11 |
| Capreomycin | *tlyA, whiB7, rrs, gidB* | 1,4,8,11 |
| Para-amnosalicylic acid | *ribD, thyA, dfrA, folC* | 5,9 |
| Cycloserine | *alr, ddl, cycA* | 6 |
| Bedaquiline | *atpE, Rv0678, mmpL5, mmpS5, pepQ,* *Rv1979c* | 2,6,12 |
| Linezolid | *rrl, rplC* | 6 |
| Delamanid | *fgd1, ddn, fbiA, fbiB, fbiC, Rv2983* | 2,6,12 |
| Clofazimine | *Rv0678, mmpL5, mmpS5, pepQ,* *Rv1979c* | 2,6,13 |

Publications used to identify these genes include

1. Ramaswamy, S. V., et al (2004). Genotypic analysis of multidrug-resistant Mycobacterium tuberculosis isolates from Monterrey, Mexico. *J Med Microbiol, 53*(Pt 2), 107-113. doi: 10.1099/jmm.0.05343-0
2. Bloemberg, G. V., et al. (2015). Acquired Resistance to Bedaquiline and Delamanid in Therapy for Tuberculosis. *N Engl J Med, 373*(20), 1986-1988. doi: 10.1056/NEJMc1505196
3. Comas, I., et al. (2012). Whole-genome sequencing of rifampicin-resistant Mycobacterium tuberculosis strains identifies compensatory mutations in RNA polymerase genes. *Nat Genet, 44*(1), 106-110. doi: 10.1038/ng.1038
4. Jnawali, H. N.,et al. (2013). Molecular genetics of Mycobacterium tuberculosis resistant to aminoglycosides and cyclic peptide capreomycin antibiotics in Korea. *World J Microbiol Biotechnol, 29*(6), 975-982. doi: 10.1007/s11274-013-1256-x
5. Mathys, V., et al. (2009). Molecular genetics of para-aminosalicylic acid resistance in clinical isolates and spontaneous mutants of Mycobacterium tuberculosis. *Antimicrob Agents Chemother, 53*(5), 2100-2109. doi: 10.1128/AAC.01197-08
6. Koser, C. U., et al (2013). Whole-genome sequencing for rapid susceptibility testing of M. tuberculosis. *N Engl J Med, 369*(3), 290-292. doi: 10.1056/NEJMc1215305
7. Manson, A. L.,et al (2017). Genomic analysis of globally diverse Mycobacterium tuberculosis strains provides insights into the emergence and spread of multidrug resistance. *Nat Genet, 49*(3), 395-402. doi: 10.1038/ng.3767
8. Sandgren, A., et al. (2009). Tuberculosis drug resistance mutation database. *PLoS Med, 6*(2), e2. doi: 10.1371/journal.pmed.1000002
9. Zheng, J., et al. (2013). para-Aminosalicylic acid is a prodrug targeting dihydrofolate reductase in Mycobacterium tuberculosis. *J Biol Chem, 288*(32), 23447-23456. doi: 10.1074/jbc.M113.475798
10. Zaunbrecher, M. A., et al. (2009). Overexpression of the chromosomally encoded aminoglycoside acetyltransferase eis confers kanamycin resistance in Mycobacterium tuberculosis. *Proc Natl Acad Sci U S A, 106*(47), 20004-20009. doi: 10.1073/pnas.0907925106
11. Dheda, K., et al. (2017). The epidemiology, pathogenesis, transmission, diagnosis, and management of multidrug-resistant, extensively drug-resistant, and incurable tuberculosis. *Lancet Respir Med*. doi: 10.1016/S2213-2600(17)30079-6
12. Bloemberg, G.V., et al. (2015). Acquired Resistance to Bedaquiline and Delamanid in therapy for tuberculosis. N Engl J Med (373):1986-8
13. Zhang, S., et al. (2015). Identification of novel mutations associated with clofazimine resistance in Mycobacterium tuberculosis. *J Antimicrob Chemother, 70*(9), 2507-2510

**Table S3: Phylogenetic markers in known drug resistance genes as per Merker et al 2020**

| **genome pos** | **gene** | **polymorphism** | **genotype association** |
| --- | --- | --- | --- |
| 7362 | Rv0006 | E21Q (gag/Cag) | non H37Rv_lab_strain |
| 7585 | Rv0006 | S95T (agc/aCc) | non H37Rv-like |
| 7892 | Rv0006 | L197L (ctg/ctA) | NEW-1 |
| 8040 | Rv0006 | G247S (ggc/Agc) | LAM (associated) |
| 9304 | Rv0006 | G668D (ggc/gAc) | non H37Rv-like |
| 575679 | Rv0486 | N111S (aac/aGc) | Haarlem |
| 575907 | Rv0486 | A187V (gca/gTa) | Beijing modern |
| 760115 | Rv0667 | D103D (gac/gaT) | Haarlem |
| 762434 | Rv0667 | G876G (ggt/ggG) | Delhi/CAS |
| 763031 | Rv0667 | A1075A (gct/gcC) | non Euro-American |
| 764995 | Rv0668 | A542A (gcc/gcG) | LAM |
| 765150 | Rv0668 | G594E (ggg/gAg) | Ghana, Haarlem, X-type |
| 766645 | Rv0668 | E1092D (gaa/gaC) | Beijing Central Asia |
| 1834177 | Rv1630 | R212R (cga/cgC) | Beijing, Beijing like |
| 1917972 | Rv1694 | L11L (cta/ctG) | non H37Rv_lab_strain |
| 2102990 | Rv1854c | V18A (gtg/gCg) | S-type (associated) |
| 2154724 | Rv1908c | R463L (cgg/cTg) | non Euro-American |
| 3073868 | Rv2764c | T202A (acc/Gcc) | LAM |
| 3840764 | Rv3423c | L219L (ctg/ctC) | H37Rv-like (associated) |
| 4238675 | Rv3792 | G248G (ggc/ggT) | Beijing Central Asia |
| 4238963 | Rv3792 | H344H (cac/caT) | S-type |
| 4239298 | Rv3792 | A456V (gcc/gTc) | Haarlem |
| 4242075 | Rv3793 | R738Q (cgg/cAg) | Delhi/CAS |
| 4242241 | Rv3793 | D793D (gac/gaT) | Beijing (associated) |
| 4242643 | Rv3793 | R927R (cgc/cgT) | non H37Rv_lab_strain |
| 4242803 | Rv3793 | V981L (gtg/Ctg) | Ghana, Haarlem, X-type |
| 4243346 | Rv3794 | Q38Q (caa/caG) | Beijing W148 |
| 4243460 | Rv3794 | C76C (tgc/tgT) | Beijing |
| 4249408 | Rv3795 | P965P (ccg/ccA) | X-type |
| 4407588 | Rv3919c | A205A (gca/gcG) | non Euro-American |
| 4407927 | Rv3919c | E92D (gaa/gaC) | Beijing |
| 4408156 | Rv3919c | L16R (ctt/cGt) | LAM |


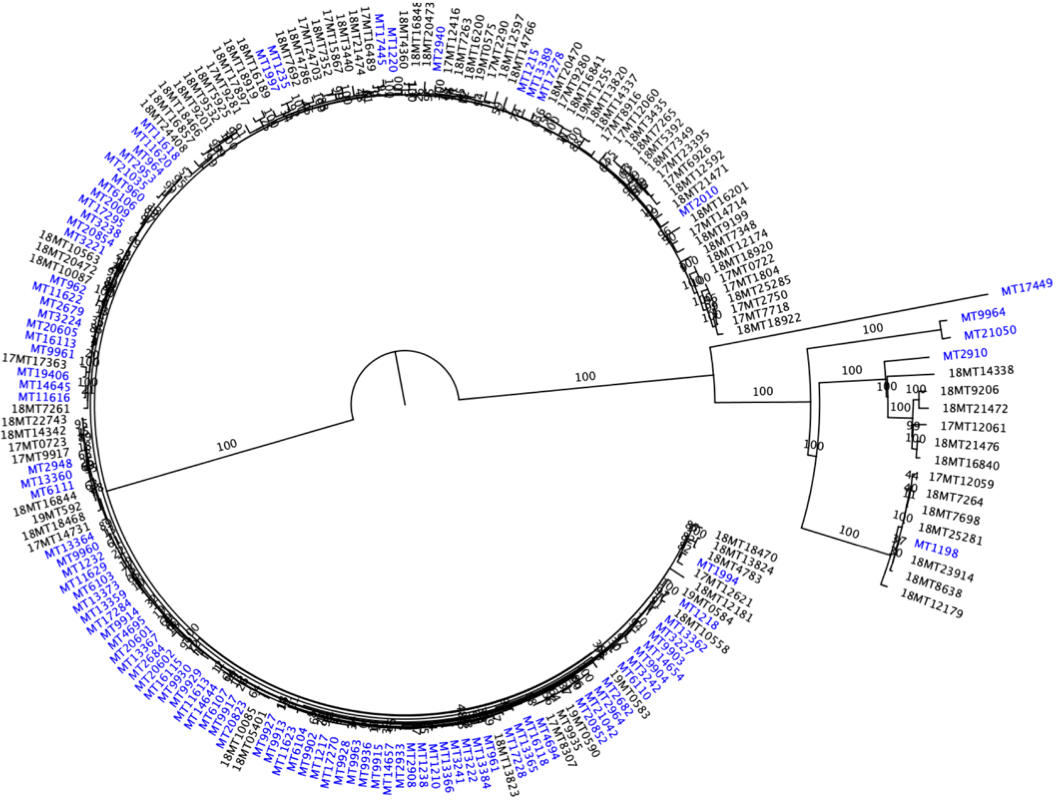


**Figure S1**: Phylogeny of 194 isolates collected from Daru (blue tip labels) and CPHL, Papua New Guinea. Maximum likelihood tree constructed using IQ-TREE, at 1000 bootstraps – GTR+I+G nucleotide substitution model based on 1809 SNPs. Two distinct lineages were observed, with majority Lineage 2 (sensi strico modern Beijing L2.2.1.1-dominant, circular branch) and Lineage 4 (Euro-American 4.8, outward branch)


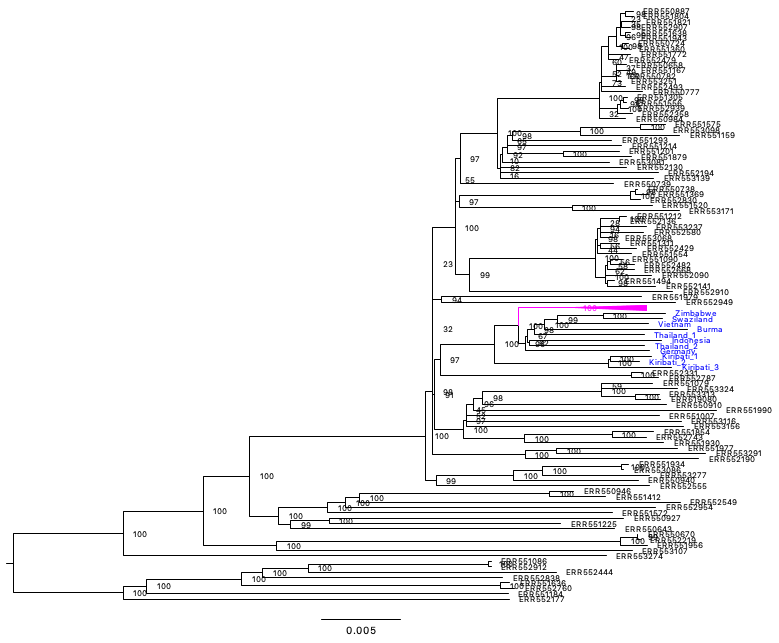


RD150

**Figure S2**: Phylogeny of 176 lineage 2 PNG isolates (purple - monophyletic clade) with a global collection of 110 Beijing genomes. Maximum likelihood constructed using IQ-TREE, at 1000 bootstraps – GTR+I+G nucleotide substitution model based on 6920 SNPs. Nearest neighbouring branch labels (blue) – show genomes identified from different countries.


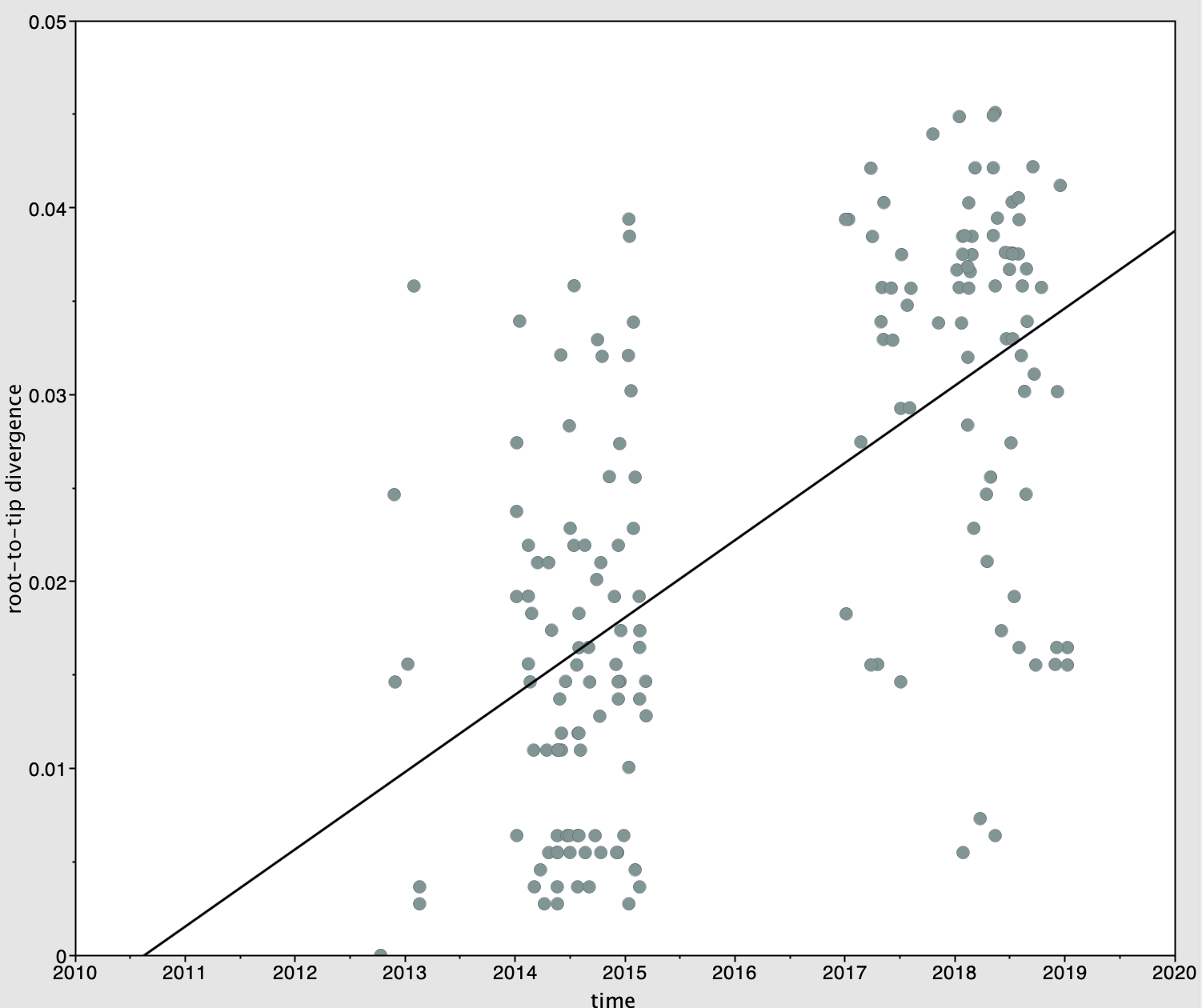


**Figure S3**: Linear regression analysis showing correlation between root-to-tip distance and sampling years (R^2^ =0.384) of an extended collection of 176 PNG Beijing datasets covering the period 2012 to 2019.

|  | **Path Sampling** | | **Stepping Stone** | |
| --- | --- | --- | --- | --- |
|  | strict | relaxed | strict | relaxed |
| No sampling date | -5527459.062 | -5527597.353 | -5527448.022 | -5527592.941 |
| With sampling date | -5527795.574 | -5527803.198 | -5527789.371 | -5527801.814 |
| log Bayes factor | 336.512 | 205.845 | 341.349 | 208.873 |

**Table S4**: Bayesian evaluation of temporal signal (BETS) results for strict and relaxed molecular clock analysis of Beijing isolates.

| **Substitution Model** | **Clock Model** | **Tree Prior** | **Path sampling** | **Stepping stone** | **Mean ESS** |
| --- | --- | --- | --- | --- | --- |
| HKY | Relaxed Lg Normal | Constant | -5527803.198 | -5527801.814 | 622 |
| HKY | Relaxed Lg Normal | Expansion | -5527785.4 | -5527770.324 | 967 |
| HKY | Relaxed Lg Normal | Exponential | -5527882.1 | -5527879.23 | 1203 |
| GTR | Relaxed Lg Normal | Skyline | -5527812.015 | -5527813.014 | 3422 |
| GTR | Relaxed Lg Normal | Constant | -5527814.816 | -5527813.062 | 1320 |
| GTR | strict | Constant | -5527814.862 | -5527814.088 | 1425 |

**Table S5:** Path and stepping stone sampling results of relaxed clock model selection based on marginal likelihood considering 100 steps and 10 million chain length.

*fabG1-inhA* (C-15T)

*inhA* (p.Ile21Val)

*ndh* (p.Glu102fs )

*rpoB* (p.Ser450Leu)

*rpoB* (p.Asp435Tyr)

*gyrA* (p.Asp94Gly)

*katG* (p.Ser315Thr)

*inhA* (p.Leu203Leu)

*fabG1-inhA* (G-17T)

*fabG1-inhA* (T-8C)

*rpoB* (p.Asp435Val)

*katG* (p.Ser315Asn)

*katG* (p.Ile364Thr)

*rpsL* (p. Lys43Arg)

*rrs* (A514C)

*rrs* (C517T)


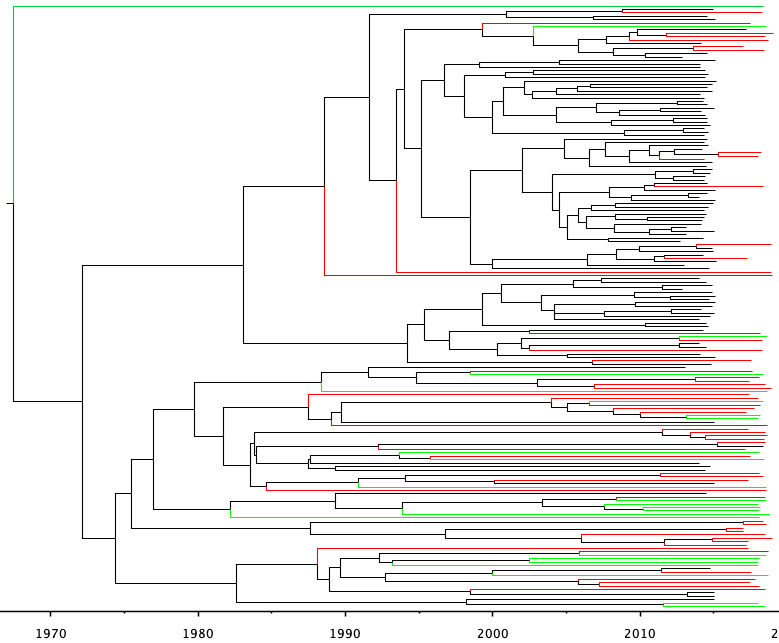


A

B

**Figure S4**: Evolutionary history of drug resistance among the Beijing strains of PNG forming two clades; A – Daru dominant clade, B – NCD dominant clade. Black tip branches – Daru isolates, red – NCD and green – isolates from other provinces. On the tree nodes are steps of fixation of different resistance conferring mutations among isolates that share the respective mutation; colors represent mutations for the different drugs, red – Rifampicin, blue – Isoniazid, Orange – Streptomycin and green – Fluoroquinolone.


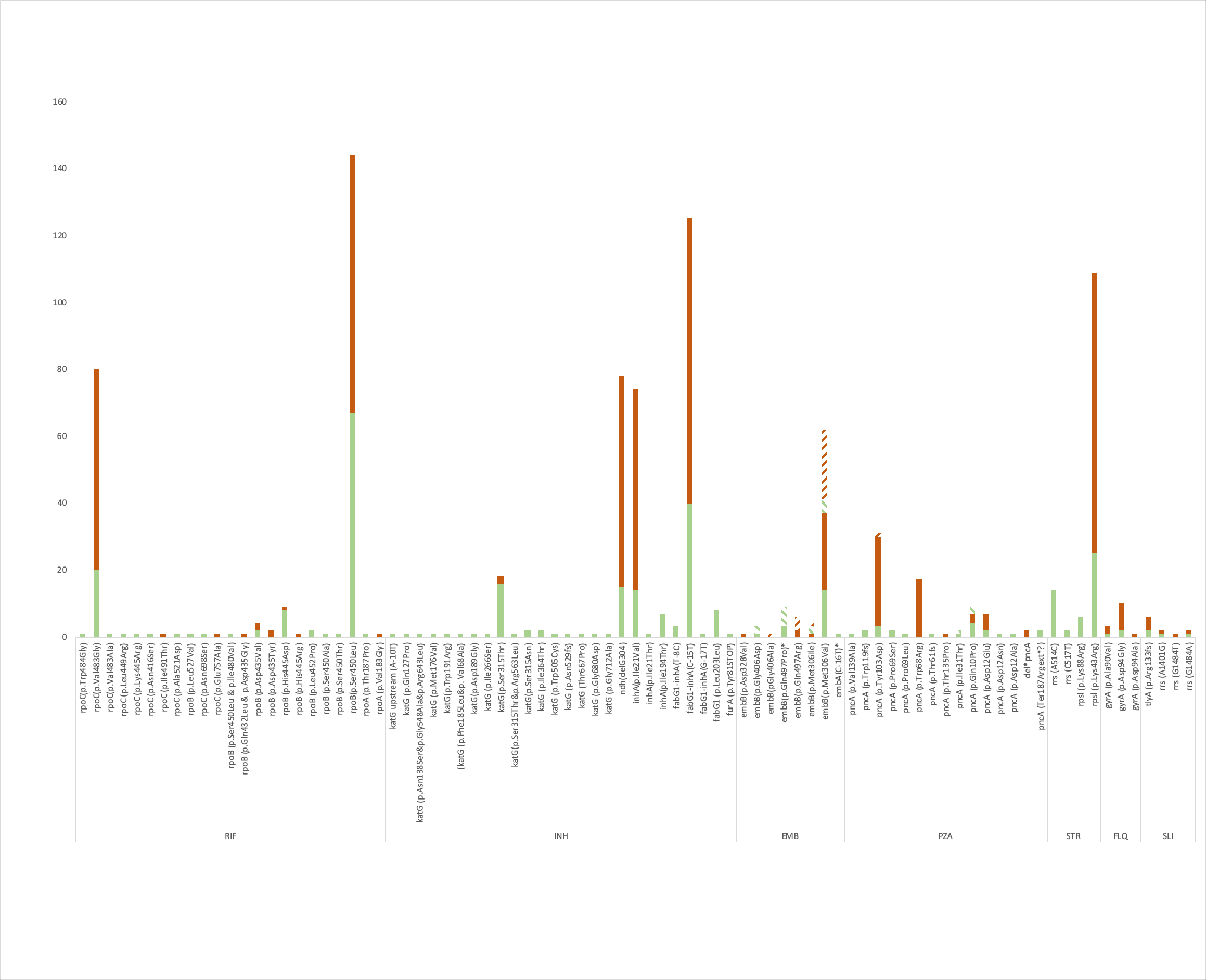


**Figure S5**: Concordance of phenotypic and genotypic drug resistance among Beijing strains from CPHL (Green – bars) and Daru (Red – bars). Some of the isolates were phenotypically susceptible but had genotypic markers for resistance, dashed green bar – CPHL, dashed red – Daru. X axis are drugs and their associated mutations, RIF – Rifampicin, INH – Isoniazid, EMB – Ethambutol, PZA – Pyrazinamide, STR – Streptomycin, FLQ – Fluoroquinolone, SLI – Second line injectables.


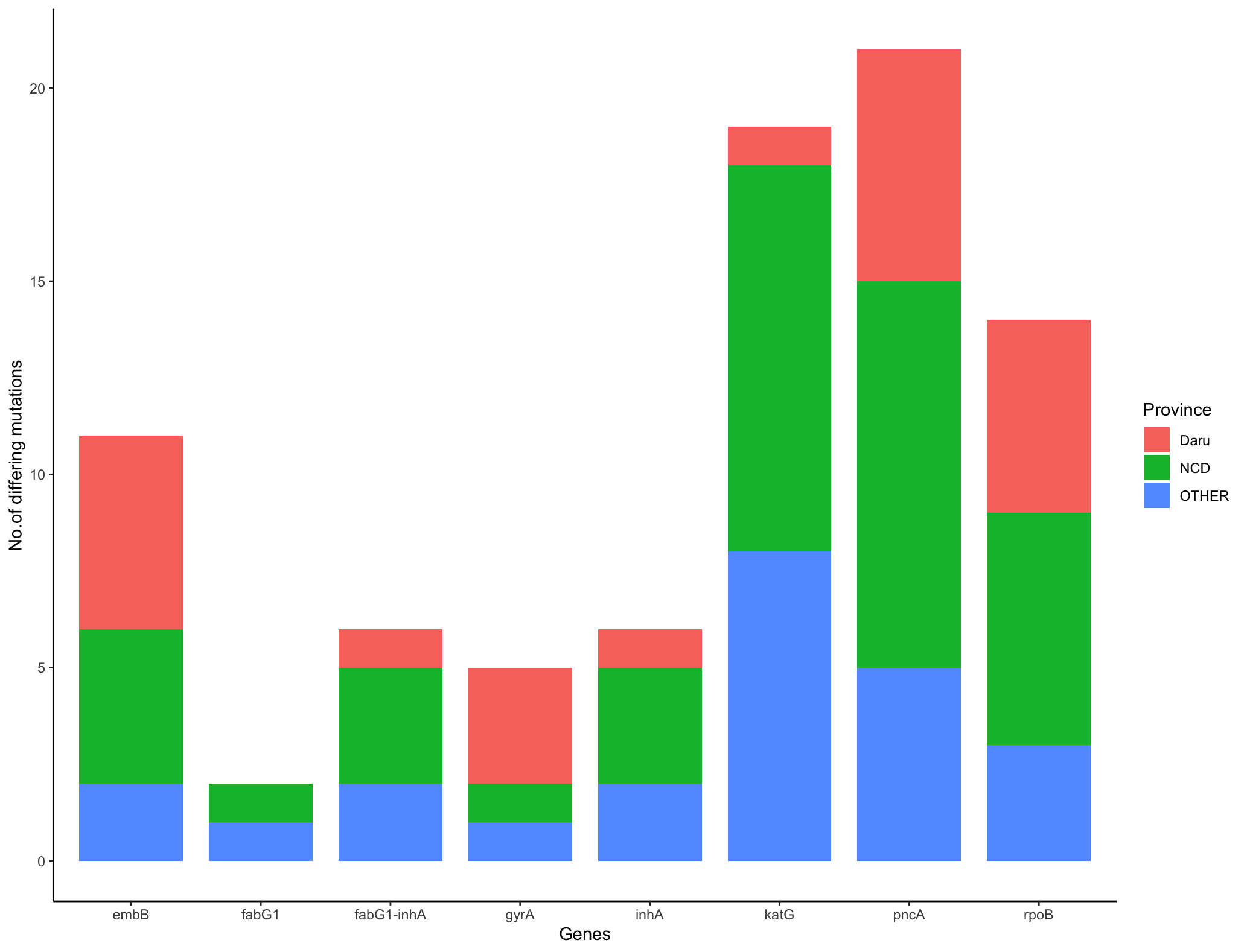


**Figure S6**: Number of unique mutations within some of the common genes known to confer resistance to First-line drugs (*embB* – Ethambutol; *fabG1*, *fabG1-inhA*, *inhA*, *katG* – Isoniazid; *pncA* – Pyrazinamide; *rpoB*- Rifampicin) and Fluoroquinolone (*gyrA*).


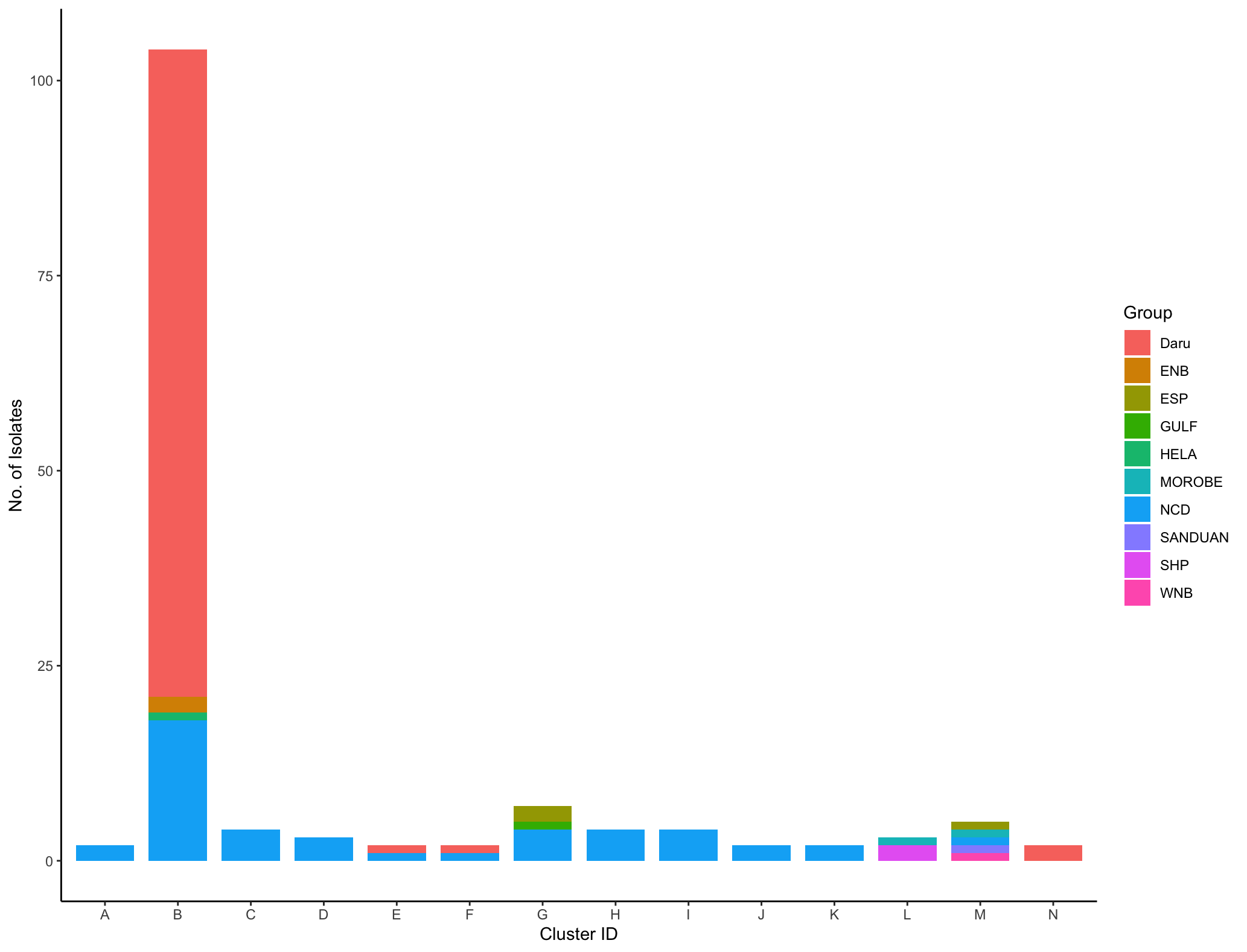


**Figure S7**: Genomic clusters created by Transcluster software (transmission threshold - 21 and transmission rate – 2) among Beijing strains, stratified by identified PNG provinces

|  |  | **Clustered** | | | | | |
| --- | --- | --- | --- | --- | --- | --- | --- |
|  |  | **Daru** | | | **NCD** | | |
|  |  | **Yes (n=87)** | **No (n=8)** | **p value** | **Yes (n=46)** | **No (n=11)** | **p value** |
| ***rpoB* compensatory mutation** | Yes | 63 | 0 | 0.0001 | 23 | 0 | 0.001 |
|  | No | 24 | 8 |  | 23 | 11 |  |
| ***inhA* promoter mutation** | Yes | 85 | 0 | 0.0001 | 31 | 3 | 0.02 |
|  | No | 2 | 8 |  | 15 | 8 |  |

**Table S6**: Association of genomic clustering among Beijing strains and two genomic features thought to be risk factors of transmission.
